# Self-Reported Empathy in Adult Women with Autism Spectrum Disorders – A Systematic Mini Review

**DOI:** 10.1371/journal.pone.0151568

**Published:** 2016-03-21

**Authors:** Francien M. Kok, Yvonne Groen, Miriam Becke, Anselm B. M. Fuermaier, Oliver Tucha

**Affiliations:** Department of Clinical and Developmental Neuropsychology, University of Groningen, Groningen, The Netherlands, and Faculty of Behavioural and Social Sciences, Grote Kruisstraat 2/1, 9712 TS, Groningen, The Netherlands; Harvard Medical School, UNITED STATES

## Abstract

**Introduction:**

There is limited research on Autism Spectrum Disorders (ASD) in females. Although the empathy construct has been examined thoroughly in autism, little attention has been paid to empathy in adult women with this condition or to gender differences within the disorder.

**Objective:**

Self-reported empathy in adult women with ASD was examined and compared to that of typically developed men and women as well as to men with this condition.

**Methods:**

Online databases were searched for articles investigating self-reported empathy among adult women with ASD. Only six studies comparing women to men were identified.

**Results:**

All studies found women with an ASD to report lower levels of empathy than typically developed women, and typically developed men, but similar levels to men with this condition.

**Conclusion:**

The self-reported empathic ability of women diagnosed with ASD resembles that of their male counterparts most closely; they show a hypermasculinisation in empathy. This is particularly surprising considering the large gender difference in empathy in the general population.

**Discussion:**

One of the limitations of this review is that the current diagnostic criteria for ASD are oriented towards male-specific behaviour and fail to integrate gender specific characteristics. Hence, women diagnosed with ASD are likely to be at the male end of the continuum. The suggested hypermasculinisation of women on the spectrum, as evident from this review, may therefore be exaggerated due to a selection bias.

## Introduction

Autism Spectrum Disorders involve social interaction and communication difficulties, as well as the presence of restricted and repetitive behaviours [1]. Findings on female-specific difficulties in these domains have mostly yielded inconsistent results. The most consistent finding has been that girls with ASD present with fewer and different repetitive and restricted behaviours than boys with this condition [2,3]. However, in the social interaction and communication domain findings remain highly inconsistent [2,4]. With a most recent estimated prevalence rate of 1.47% [5], ASDs are relatively common and they are described as becoming increasingly prevalent [5–7]. ASDs are approximately five times less prevalent among women (1 in 189) than among men (1 in 42) [5]. The female:male ratio for high functioning ASD is even lower at approximately 1:10 [8,9], whereas in low IQ samples this gender gap narrows considerably [8,10]. Although there is evidence of an overestimation of this gender bias in autism diagnosis due to diagnostic / ascertainment differences [11–14], a gender ratio of at least 2:1–3:1 remains [15]. Several reasons for the lower rate of autism diagnosis in women have been suggested. First, it has been suggested that women with ASD may be protected against some of the impairments associated with this disorder; also referred to as the female protective effect (FPE) [16,17]. The FPE suggests that a) female gender protects girls from autistic impairments, and b) in order for girls to manifest impairments associated with ASD they require a greater etiologic load than boys. This protective factor is sufficiently strong to prevent a small to medium level of genetic disruption from causing impairment in girls, but not a large level, explaining the tendency for girls with a diagnosis of ASD to present with more severe symptoms than boys [7,16–18]. Second, evidence exists that women with an ASD have a distinct, female phenotype altogether different from that of men [19]. This phenotype is likely to be detected by current diagnostic criteria and screening instruments less frequently because current criteria have been derived from studies involving predominantly male samples and are therefore likely to be male-specific [20]. Third, the female ASD phenotype may be particularly difficult to identify in women with average to above average cognitive level, as they are better able to camouflage their symptoms [21]. Fourth, there may simply be a higher occurrence of autism in males due to the difference in male hormones between men and women. For example, studies have found an association between exposure to increased levels of fetal testosterone and the development of ASD and autistic traits (i.e. the androgen theory of autism) [22–29], leading to the Extreme Male Brain (EMB) Theory of Autism [30]. According to this theory, certain prenatal factors, such as the exposure to increased levels of testosterone in utero [27,30,31], may contribute to the development of a hypermasculinised brain. This theory is supported on a behavioural level by the empathising-systemising (E-S) theory of autism [30,32], which is the focus of the present paper.

The Empathising-Systemising (E-S) theory distinguishes two independent cognitive styles, empathising and systemising, which are thought to be differentially pronounced in women as compared to men. Women are believed to be more apt at empathising, which describes the ability to accurately identify another person’s mental state and to respond to it with appropriate emotions of one’s own. Men, on the other hand, are thought to be more apt at systemising, which is the ability to analyse and construct systems whereby allowing the individual to discover regularities in this system’s ‘behaviour’ and consequently predict how it will behave in the future. Baron-Cohen and colleagues [33] proposed that people with ASD show average to superior systemising together with diminished empathising; i.e. the stereotypically male cognitive style. This led to the extension of E-S Theory to the, in the previous paragraph mentioned, the Extreme Male Brain (EMB) Theory of Autism [30]. The development of a hypermasculinised brain may in turn lead to the cognitive style of increased systemising and decreased empathising [23]. Further, people with ASD may present with an ‘extreme male brain’ in the quite literal sense [23]: neuroimaging studies suggest that brain regions which tend to be larger in typically developed males than in females, such as the cerebellum and amygdala, are yet larger in those with an ASD. Likewise, regions which are usually smaller in males than in females with typical development (e.g. prefrontal cortex, thalamus, anterior cingulate, and parts of the temporal gyrus) tend to be even smaller among people with ASD. In line with the Extreme Male Brain Theory, people on the spectrum consistently report lower levels of empathy [30,32,34–39].

Empathy has been measured by several self-report instruments, such as the Interpersonal Reactivity Index [40] and Bryant's Empathy Index [41] in persons with ASD. The studies in the present review all used the Empathy Quotient (EQ) [42], a self-report questionnaire developed to measure empathy through forced-choice items. The EQ is the most frequently used and most validated instrument for measuring empathy in ASD [43], and its advantages are that it is short and both easy to complete and to score [44]. Using this instrument, people with an ASD have been reliably found to endorse lower levels of empathy than typically developed individuals, with typically developing women reporting the highest levels of empathy, followed by typically developing males [30,32,34–38,45]. Similarly, the systemising aspect of E-S Theory has also been assessed by means of self-report: the Systemising Quotient [45]. When combining the results of both questionnaires, different ‘brain types’ can be determined. Extreme Type E brain (E>>S) is assigned to those who score very high on the EQ and low on the SQ. In cases where scores on the EQ are higher than those on the SQ, a person is said to be of Type E (E>S), whereas similar scores on both EQ and SQ yield Type B (balanced; E = S). Similarly, Extreme Type S brain (S>>E) is assigned to those who score very high on the SQ and low on the EQ, and Type S (S>E) to those whose scores on the SQ are higher than those on the EQ. Using these brain types, Baron-Cohen et al. [34] demonstrated that, in addition to reduced levels of empathy, ASD is related to enhanced systemising, with more individuals with ASD showing systemising or extreme systemising brain types. Although many studies have investigated gender differences in empathy among the general population [46,47], research on empathy among women with an ASD is scant. Despite the lack of research on the presentation of ASD among women, some interesting findings have been yielded [29]. When compared to males, females with this condition were found to be better able to integrate verbal and non-verbal behaviours [13,48], maintain reciprocal conversation [48,49] and initiate friendships [48] but not maintain these [50,51]. They generally present with less typically autistic traits [52], considerably less and different restricted interests [2,48,50,53,54], less comorbidity [55,56], and experience less problems at school [2]. On the other hand, women have been found to show more sensory symptoms [21,57], greater impairments in empathic behaviour as toddlers [58], and more extensive social deficits overall [54,58], although studies have also found higher self-reported [42] and teacher-reported social functioning [2]. This leads us to believe that the willingness or ability to maintain social ties may be differentially affected among women with autism.

It has been suggested that these reported gender differences in social behaviour can be ascribed to traditional gender roles [51,59], as women in general are expected to show stronger social skills than men. These dissimilar expectations may lead parents and others in the girl’s surrounding to recognise and appraise their symptoms differently. When the social skills of girls with ASD are impaired compared to those of typically developing girls, they may still be similar to or even better than those of typically developing boys. This may lead to a missed diagnosis due to incorrect appraisal of social impairment in girls. Alternatively, the restricted or repetitive behaviours evidenced by girls with autism may revolve around social encounters [29,60]. Girls with autism have, for instance, been described as keen observers of other children’s play and may frequently imitate the actions of those around them [19,60]. As a result of this imitation, they may display more ‘socially appropriate’ behaviour and camouflage alterations in their conduct. Despite clear gender differences in empathy in the general population, the majority of existing studies treat people with an ASD as a homogeneous group. While this may be largely owed to the supposedly lower prevalence among women and the resulting small number of women with this condition included in most studies, this situation does not offer the best possible test of either E-S or EMB Theory. According to EMB Theory, hypermasculinisation would lead the empathic ability of women with an ASD to resemble that of men more closely than that of other women. The purpose of this review is thus to investigate self-reported empathy in adult women on the spectrum and to delineate how they differ from their male counterparts in their self-reported empathy. In doing so, it will be of particular interest to determine whom these women resemble most closely: typically developed women, typically developed men, or men with an ASD. With this study we hope to raise awareness for female-specific autism, as gender differences have long been neglected in ASD research and in psychiatry in general, as well as highlight the issue of male biased ASD diagnostics.

## Method

**Fig 1:**
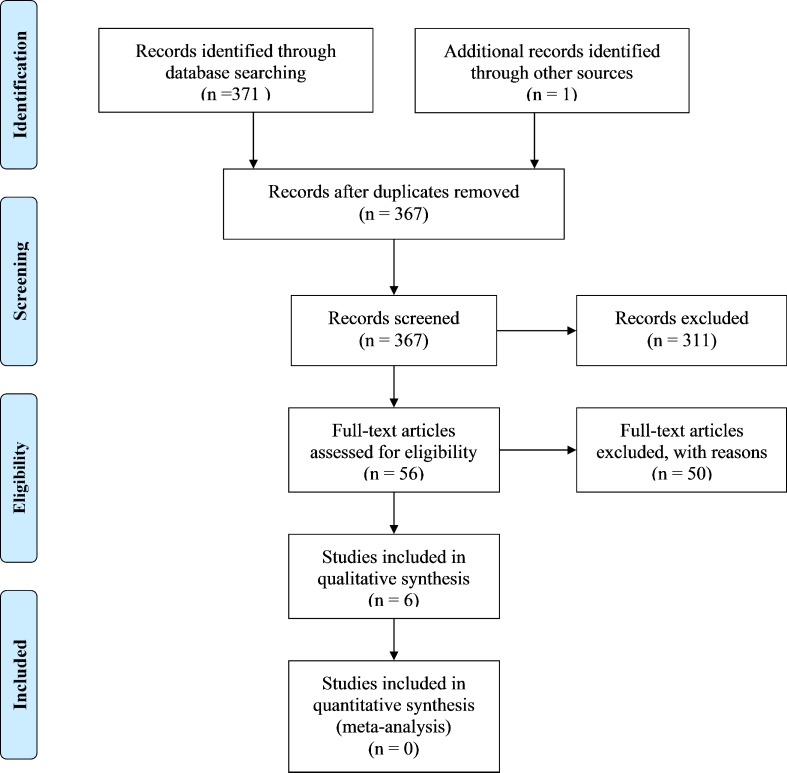
PRISMA 2009 Flow Diagram.

This systematic literature review was performed conforming to the guidelines of Preferred Reporting Items for Systematic Reviews and Meta-Analyses (PRISMA) (see S1 Table for a checklist of the PRISMA guidelines for this study). Several procedures were used to identify potential studies for this review. First, the literature was searched electronically in PsychINFO, PubMed, and Web of Knowledge including all of the available literature up until the date of October 10, 2015. The primary keywords ‘autism’, ‘ASD’, ‘Asperger’s’, and ‘PDD-NOS’ were combined with the keyword ‘empathy’ as well as with ‘female’, ‘sex’ and ‘gender’. In order to be included in this review, studies had to meet the following inclusion criteria: (a) individuals with an ASD rated their levels of empathy by means of self-report, (b) the study included an adult sample (i.e. participants of age 18 or older), (c) both a female and a male sample was included, and (d) studies were published in English. Finally, reference lists of relevant studies included in the present review were used to locate additional studies. Initial search yielded 371 records; after removal of duplicates, screening and exclusion of irrelevant articles, six studies were included in the qualitative synthesis. Most articles were excluded for not including both a sample of women and a sample of men with ASD, for not using a self-report measure, or for including a sample of only children and / or adolescents (see Fig 1 for a flow diagram of this process).

### Identified Outcome Measures

The studies in this review all used the EQ [42] as self-report measure of empathising, and five of the six studies used this in combination with the SQ [45] as measure of systemising.

In those studies where both the EQ and SQ are administered, comparison of the resulting scores allowed for the determination of the previously described ‘brain type’ and other derived scores. The D Score [35] could be calculated by subtracting standardised scores on the EQ from those on the SQ and dividing the result by two. A high resulting score indicates strong systemising in the face of poor empathising, or low capabilities in both domains. Adding scores on the EQ and SQ and dividing the result by two yielded a second derived score, the C Score [35]. This score quantifies the reciprocal relationship between empathising and systemising, such that it indicates whether one score increases while the other decreases.

In order to investigate self-reported empathy in women with an ASD and make comparisons to men with this condition as well as to typically developed adults, group means on the EQ were compared between women and men with and without an ASD. Further, effect sizes (Cohen’s *d*) yielded by the comparison of group means were investigated. The weighted average of effect sizes per group was computed by weighing each study by the number of participants (*n*), giving more weight to larger, and by this more robust studies. Women with an ASD (FASD) were compared to men with this condition (MASD), to typically developed women (FTD), and to typically developed men (MTD). Further comparisons were made between typically developed women (FTD) and typically developed men (MTD), men with an ASD (MASD) and typically developed men (MTD).

## Results

Most studies [13,14,16,26,27] found women with an ASD not to differ from their male counterparts in self-reported empathy, and the group as a whole exhibited lower scores on the EQ than typically developed men. Results on the EQ per study are presented in Table 1, and more details can be found in S1 Table.

**Table 1:** Group Means and Standard Deviations on Empathy Quotient by Study.

Study	*n* (% of females)	ASD		*n* (% of females)	TD	
		F	M		F	M
**Baron-Cohen (2014)[34]**	811 (56.0)	26.4 (17.2)	20.4 (12.4)	3906 (65.6)	48.5 (13.7)	38 (13.7)
**Goldenfeld (2005)[35]**	47 (29.8)	23.4 (14.1)	18.9 (9.9)	278 (59.0)	47.7 (11.0)	38.8 (12.4)
**Lai (2011)[21]**	62 (46.8)	20.1 (10.9)	18.9 (7.6)	NA	NA	NA
**Sucksmith (2013)[36]**	329 (51.1)	18.2 (8.9)	17.5 (10.5)	187 (50.3)	48.5 (14.1)	37.7 (13.5)
**Wakabayashi (2007)[37]**	48 (20.8)	26 (7.83)	24.6 (8.53)	137 (48.2)	36.9 (10.37)	31.1 (10.7)
**Wheelwright (2006)[38]**	125 (44.8)	18.5 (10.1)	18.7 (9.8)	1761(58.9)	48 (11.3)	39 (11.6)

*Note*. ASD–Autism Spectrum Disorder; NT–Neurotypical (typically developed); F–Female; M–Male; NA–Not Applicable.

One study [34] found higher self-reported empathy in women on the spectrum compared to their male counterparts. The effect size for this comparison (d = 0.393) was, however approximately half of the corresponding comparison in the typically developed group (d = 0.767). Inspection of the remaining respective effect sizes further substantiates this pattern (see Table 2). All six studies found evidence for diminished self-reported empathy among adults with an ASD compared to typical women and men. Significant gender differences were demonstrated in the general population, with women evidencing higher levels of empathy than men. Within the clinical group, gender differences were either diminished or entirely abolished. The smallest effect sizes are found when comparing women and men with an ASD, suggesting that self-reported levels of empathy are most alike in this group. Indeed, this comparison yields a much smaller weighted average effect size (d = 0.264), approximately one-eighth of that between females on the spectrum and those with typical development (d = 1.944). Women and men with an ASD are further more alike (d = 0.264) than typically developed men and women (d = 0.766). Lastly, women on the spectrum report levels of empathy more akin to those of typically developed men (d = 1.136) than women with typical development (d = 1.944).

**Table 2:** Effect Sizes Yielded by Comparison of Group Means on Empathy Quotient.

Study	FASD-MASD	FTD-MTD	FASD-FTD	MASD-MTD	FASD-MTD
**Baron-Cohen (2014)[34]**	0.393	0.767	1.548	1.310	0.792
**Goldenfeld (2005)[35]**	0.408	0.770	2.171	1.685	1.233
**Lai (2011)[21]**	0.131	NA	NA	NA	NA
**Sucksmith (2013)[36]**	0.072	0.787	2.754	1.736	1.819
**Wakabayashi (2007)[37]**	0.170	0.554	1.094	0.656	0.496
**Wheelwright (2006)[38]**	0.020	0.788	2.626	1.774	1.785
**Weighted average**	0.264	0.766	1.944	1.446	1.136

*Note*. ASD–Autism Spectrum Disorder; TD–Typically developed; F–Female; M–Male; NA–Not Applicable.

As participants were administered the SQ in addition to the EQ as part of some studies [34–37], the distribution of brain types could be identified, and comparisons between the genders can be made, as well as between typically developed people and those on the autism spectrum (see Table 3; S1 Table). As most studies did not present data on women separately, instead using a clinical ‘ASD’ group, brain types of women on the autism spectrum in particular are only highlighted in one study [34].

**Table 3:** Most Common Brain Types Identified per Group and their Respective Frequency.

Study	Brain Types (% identified) per group		
	ASD	FTD	MTD
**Baron-Cohen (2014)[34]**	Females	Type E (43.1%)	Type S (53.7%)
	Type S (46.8%)	Type B (30.6%)	Type B (28.4%)
	Extr. Type S (27.4%)		
	Males		
	Type S (60.3%)		
	Extr. Type S (31.3%)		
**Goldenfeld (2005)[35]**	Extreme Type S (46.8%)	Type E (44.2%)	Type S (53.5%)
	Type S (40.4%)	Type B (35.0%)	Type B (23.7%)
**Wakabayashi (2007)[37]**	Type S (36.8%)	Type B (42.4%)	Type B (49.3%)
	Extreme Type S (31.6%)	Type E (28.8%)	Type S (22.5%)
**Wheelwright (2006) [38]**	Extreme Type S (61.6%)	Type E (44.8%)	Type S (49.5%)
	Type S (32.0%)	Type B (29.3%)	Type B (30.3%)

*Note*. ASD–Autism Spectrum Disorder; FTD–Typically developed women; MTD–Typically developed men; F–Women; M–Men.

A high prevalence of Extreme Type S and Type S among individuals with an ASD was found in all studies [34,35,37,38]. Notably, both Type E and Extreme Type E were either observed at very low levels, or not at all, in these clinical groups. This pattern was unique to ASD, as typically developed women in these studies most commonly evidenced Type E, followed by Type B. Typically developed men most often gave responses in line with type S, followed by Type B. The authors reported a high prevalence of the balanced type within the general population, which was not as pronounced among individuals with an ASD.

The large study by Baron-Cohen et al [34] was the only study to use a female ASD as well as a male ASD group, rather than a combined clinical group. Among women on the autism spectrum, Type S was most common (46.8%), followed by Extreme Type S (27.4%), Type B (12.6%), Type E (11.6%) and Extreme Type E (1.5%). In men of this group the order of incidence of brain types was the same as in women, but the number of individuals showing either Type S or Extreme Type S was higher, and Extreme Type E was not observed. Gender differences were thus confirmed in the clinical group, where women were more likely than their male peers to evidence a Type E or Type B profile. Further, this study demonstrated that women with an ASD showed a higher preference for systemising than typically developed men and women.

Using the D Score, the finding of stronger systemising than empathising among individuals with an ASD may be further illustrated [34,35,38]. Women with ASD had lower D scores, reflecting a more empathic cognitive style, than men with this condition, but this gender difference was much less pronounced than in the typically developed group. People in the clinical group overall evidenced higher D scores than both women and men with typical development, providing additional, numerical evidence of strong systemising abilities in light of weaker empathising tendencies in this clinical group. Moreover, no gender differences were found in C Scores among women and men with typical development [35]. This is taken as evidence of ‘competition’ between empathising and systemising in the neurotypical brain: women and men who developed typically may, on average, differ in their empathising and systemising abilities, but there are no differences in ability when the two cognitive styles are considered as a whole. Differences were, however, found between the typically developed and the clinical group, wherein people on the spectrum tended to show lower C scores than those with typical development. This suggests that the stronger systemising ability seen in people with autism may not suffice to compensate for their lower empathising ability.

## Discussion

The purpose of this review was to examine self-reported empathy in adult women with an autism spectrum condition, and to shed light on how they differ from their male counterparts, in order to raise awareness for female-specific autism and male biased ASD diagnostics.

### Conclusions

The scarce existing body of literature of only six studies unequivocally suggests that women with ASD report similar levels of empathy to men with this condition; that is, their empathy levels are equally reduced. Gender differences, as they are found using the EQ and other instruments in the general population, are diminished [34] or abolished in autism spectrum conditions [36,37,61]. Women with ASD report much lower levels of empathy than typically developed women. In this respect, women with ASD resemble men with this condition more so than typically developed women. The difference in empathy levels between women with and without ASD was larger (Cohen’s d = 1.9) than that between men with and without ASD (Cohen’s d = 1.4), indicating that women are indeed more affected by ASD than men. This large difference between clinical and non-clinical women also demonstrates the importance of including a female control group when investigating female specific ASD characteristics. Although in the clinical group women were similar to men, they were significantly more likely to present with brain Type E or Type B than their male counterparts [34]. However, most of them indicated Extreme Type S or Type S brain types, and very few regarded themselves Type E or Extreme Type E. Notably, they were twice as likely as typically developed women to endorse answers in line with brain Type S, and showed higher preference for systemising than typically developed men. It must be noted that the superior systemising abilities found in people with autism may not suffice to compensate for the diminished tendency to empathise [35]. Lastly, although women with ASD had lower D scores than their male counterparts, their D scores were much higher than those of the typically developed women, reflecting a more systemizing cognitive style. The suggested hypermasculinisation of women with autism is supported insofar as that the women included in the samples examined in the present review did not only resemble men with autism, but also typically developed males, more closely than other women.

### Limitations

One serious limitation of the reviewed studies is that they likely suffer from different forms of selection bias. First, all studies included in this review are from the Baron-Cohen workgroup and associated scientists. Over 50% of total participants are taken from one publication [34], and over 75% from two publications [34,36]. Moreover, the majority of other participants were collected in the Cambridge area. It is advisable that independent research groups replicate these findings. Second, what requires further mentioning is the presence of a very large standard deviation in the female ASD group of the biggest sample [34], which may put question marks over the online selection of women who report to have a clinical diagnosis of ASD. Although all participants in the ASD group reported a clinical diagnosis of ASD, these diagnoses could not be verified because data were collected online. Third, the reviewed studies may suffer from a selection bias of diagnosed women with ASD according to male-based criteria, which may have led to an exaggeration of the evidenced hypermasculinisation of empathy. There is currently a strong male bias in the diagnosis of ASD, because the international diagnostic criteria are based on studies that primarily included male samples [1,28], as a result of the higher prevalence of autism in males compared to females [5]. It is likely that women who do not present with male-specific ASD symptoms are being overlooked entirely, as the clinical presentation of ASD in women is not yet clear. Several studies have attempted to characterise the female ASD phenotype and proposed the development of gender-specific diagnostic criteria [54,57,62]. Kopp and Gillberg [62] are the first authors to have published a female-specific ASD screening instrument and distinguished both a female and a male ASD type. Currently, those women who do receive a clinical diagnosis of ASD are likely to present with male-specific ASD symptoms. Further, research has shown that girls with comparable levels of autistic traits to boys tend to not receive a diagnosis of ASD unless they also present with cognitive impairment or behavioural problems [63]. Diagnosed girls or women with ASD may therefore well be on the more severely affected end of the ASD continuum. This converges to this review’s finding that women with ASD report hypermasculinised empathy, which is at odds with the medium to large gender difference in empathy in typically developed individuals [43]. Research is therefore needed attempting to integrate female specific characteristics in the definition of ASD. This may lead to the formalisation of gender specific symptoms and behaviour in ASD (qualitative aspects) [13,64,65] but also to the recommendation of gender specific cut-offs when applying clinical questionnaires and rating scales (quantitative aspects) [62].

Another limitation, inherent to all self-report measures, is that socially desirable responding may be a concern with all described studies and hinder the interpretation of findings [66]. Conceivably, groups may have been differentially affected by this response bias. Pressures conveyed by traditional gender roles may have led women to respond in line with the expectation of them to show stronger empathising than males [67]. Adherence to traditional gender roles may consequently have both altered the way in which women and men interpreted the items and artificially increased the differences between the genders [68]. It is further possible that participants' diagnostic status influenced their susceptibility to the social desirability bias and therefore their responding. It has been suggested that people with an ASD may be less inclined to behave in a socially desirable manner than typically developed individuals [69]. If this was also the case in the presently reviewed studies, the differences between the typically developed and ASD groups may have been inflated. On the other hand, if individuals with an ASD overestimated their true ability due to subpar insight, this may have resulted in the reported differences being smaller than the true ones [70]. Moreover, a difference with regards to social motivation might exist between the different ASD subtypes [69,71]. For example, individuals with ASD who are socially motivated might be more affected by response bias than those who are not, and that this might be differentially represented in males and in females.

### Outlook for Future Research

The present review adds to the limited body of knowledge on women with ASDs, but extensive research is needed in order to shed light on the differential presentation of women with this condition and thereby improve their identification. At present, clinicians are only beginning to become aware of gender-based ASD profiles. We therefore call for future studies using female samples, to elucidate the female profile of autism and develop gender norming for the entire diagnostic process. To the authors’ knowledge, only one such study has been carried out, involving 71 girls with ASD and 58 TD girls, by Kopp and Gillberg [62] in an attempt to revise an ASD screening questionnaire in order to better capture the female ASD phenotype. Their study demonstrated that certain single questionnaire items could be used in the diagnostic process of ASD in girls. However, multiple studies involving large samples of females are needed. Further, the present review only included studies using a self-report measure of empathy. This is a subjective measure, and therefore it would be interesting to demonstrate whether these results can be replicated using both subjective and objective measures of empathy in ASD. For example, social-cognitive tasks (such as the Eyes task [72] or the Empathic Accuracy task [73], as well as physiologic measures [74] could be used. Moreover, in the present review, empathy is viewed as a unitary construct, even though it has been suggested that the EQ measures three different types: cognitive empathy, affective empathy, and social skills. The dissociation of cognitive and affective empathy is of particular interest, as gender differences may be more pronounced in affective compared to cognitive empathy [43]. Herein, cognitive empathy refers to the ability to infer another person's state of mind, whereas affective empathy describes an individual’s appropriate emotional reactions to another person's state of mind. This distinction between cognitive and affective empathy can be made based on subscales of the EQ and allow for a more differentiated account of gender and group differences in self-reported empathy. Differentiating between cognitive and affective empathy by examining the EQ’s subscales separately in future studies could prove useful in shedding light on these different possibilities.

## Supporting Information

S1 File (DOCX)

S1 Table PRISMA Checklist. (DOC)
