# Supplementary material for: Self-Reported Empathy in Adult Women with Autism Spectrum Disorders – A Systematic Mini Review
Source: PLoS One. 2016 Mar 21;11(3):e0151568. doi: 10.1371/journal.pone.0151568 (PMC4801394; doi:10.1371/journal.pone.0151568)
Supplement: S1 File — (DOCX) [file pone.0151568.s001.docx]

**Supporting Information.**

Table 1. Summary of Main Findings per Study

| **Authors (year)** | **N (% female)** | **Age (*SD*)** | **IQ (*SD*)** | **Outcome Measure(s)** | **Main findings** |
| --- | --- | --- | --- | --- | --- |
| **Baron-Cohen (2014)** | ASD: 811(55.98)  TD:  3906 (65.59) | F: 34.5 (13.1)  M: 34.9 (13.3)  F: 34.4 (12.5)  M: 34.4 (14.3) | N.R. | EQ, SQ | EQ: ASD < TD; F < M in both groups (*d*=0.76 in NT group, *d*=0.4 in ASD group)  D Score: ASD > TD_M_ > TD_F_ |
| **Goldenfeld (2005)** | ASD: 47(29.79)  TD: 278(58.99) | N.R. | N.R. | EQ, SQ | D Score: ASD > TD_M_ > TD_F_ C Score: ASD < TD_M_ = TD_F_ |
| **Lai (2011)** | ASD: 62(46.77) | F: 26.9 (6.7)  M: 27 (7.1) | F: 112.8 (15.7)  M: 112.6 (16.3) | EQ | ASD_F_ = ASD_M_ |
| **Sucksmith (2013)** | ASD: 329(51.06)  TD: 187(50.27) | 35.5 (11.03) 34.2 (10.76) | 53.7 (3.58) 52.3 (4.24) | EQ | ASD_F_ = ASD_M_ < TD |
| **Wakabayashi (2007)** | ASD: 48(20.83)  TD: 137(48.18) | 28.9 (8.92) 29.6 (4.46) | 96.8 (17.97)  N.R. | EQ | ASD_F_ = ASD_M_ < TD_M_ < TD_F_ |
| **Wheelwright (2006)** | ASD: 125(44.80)  TD: 1761(58.94) | 37.6 (13.1)  21 (2.58) | N.R. | EQ, SQ | EQ: ASD_F_ = ASD_M_  D score: ASD > TD_M_ > TD_F_ |

*Note*. ASD – Autism Spectrum Disorder; TD –Typically developed; F – Female; M – Male; EQ – Empathy Quotient; SQ – Systemising Quotient; N.R. – Not Reported.

Table 2. Prevalence Rates of Main Findings per Study.

| **Study** | **Group** | **Extreme E** | | **E** | | **B** | | **S** | | **Extreme S** | |
| --- | --- | --- | --- | --- | --- | --- | --- | --- | --- | --- | --- |
|  |  | F | M | F | M | F | M | F | M | F | M |
| **Baron-Cohen** | ASD | 1.5 | 0 | 11.6 | 1 | 12.6 | 7.4 | 46.8 | 60.3 | 27.4 | 31.3 |
|  | TD | 3.7 | 0.3 | 43.1 | 12.9 | 30.6 | 28.4 | 21.2 | 53.7 | 1.4 | 4.7 |
| **Goldenfeld (2005)** | ASD | 0 | 0 | 0 | 0 | 12.8 | 12.8 | 40.4 | 40.4 | 46.8 | 46.8 |
|  | TD | 4.3 | 0 | 44.2 | 16.7 | 35 | 23.7 | 16.5 | 53.5 | 0 | 6.1 |
| **Wakabayashi (2007)** | ASD | 0 | 0 | 2.6 | 2.6 | 28.9 | 28.9 | 36.8 | 36.8 | 31.6 | 31.6 |
|  | TD | 16.7 | 0 | 28.8 | 11.3 | 42.4 | 49.3 | 9.1 | 22.5 | 3 | 16.9 |
| **Wheelwright (2006)** | ASD | 0 | 0 | 0 | 0 | 6.4 | 6.4 | 32 | 32 | 61.6 | 61.6 |
|  | TD | 4.3 | 0.1 | 44.8 | 15.1 | 29.3 | 30.3 | 20.7 | 49.5 | 0.9 | 5 |
| ***Mean*** | ASD | 0.4 | 0 | 3.6 | 0.9 | 15.2 | 13.9 | 39 | 42.4 | 41.9 | 42.8 |
|  | TD | 7.3 | 0.1 | 40.1 | 14 | 34.3 | 32.9 | 16.9 | 44.8 | 1.3 | 8.2 |

*Note*. ASD – Autism Spectrum Disorder; TD – Typically developed; F – Female; M – Male.
